# State Association of Druggists

**Published:** 1879-06

**Authors:** 


### State Association of Druggists.

The Druggists of the State, to the number of about ——, met in Utica, May 21st, to form a State Association.

Prof. BEDFORD said: In calling this meeting to order, the idea of an Association of Druggists of the State is not a new one. Such Associations exist in seven of the States, and in each have elevated the status of Pharmacists. Their organization dates back twenty-seven years when the American Pharmaceutical Association was formed in New York. This stands in the same relation to Druggists as the American Medical Association does to Physicians.

After the adoption of a Constitution and By-Laws, the following officers were chosen for the ensuing year.

*President*—Prof. P. W. Bedford, New York; *First Vice-President*—C. M. Lyman, Buffalo; *Second Vice President*—Benjamin F. Ray, Utica; *Third Vice-President*—A. J. Inloes, Binghamton; *Secretary*—Charles H. Gaus, Albany; *Assistant Secretary*—Clay W. Holmes, Elmira; *Treasurer*—William Blaikie, Utica.

*Executive Committee.*—Louis E. Nicol; Brooklyn; A. B. Husted, Albany; E. H. Davis, Rochester.

*Delegates to American Pharmaceutical Association*—F. F. Knapp, New York, W. H. Rogers, Middletown; C M. Lyman, Buffalo; William Blaikie, Utica; H. P. Napier, Owego.

The next meeting will be held at Syracuse, the 3d Wednesday in May, 1880.

